# Reimagining Music for Older Adults: Fresh Ideas and Bold Possibilities

**DOI:** 10.1093/geroni/igaf122.706

**Published:** 2025-12-31

**Authors:** Lisa Lehmberg, Victor Fung, Justine McGovern

## Abstract

In this interdisciplinary symposium, attendees are invited to “think outside the box” as new avenues regarding the role of music in older adult well-being and quality of life are explored via four interconnected research presentations spanning philosophy, education, social science, and health. The first presentation establishes a theoretical foundation through classic Daoist and Confucian perspectives, offering philosophical analyses into music’s capacity to foster harmony, balance, and societal well-being. Building on this, the second presentation shares empirical research findings on intergenerational music-making as a means to counteract ageism, strengthen social bonds, and support lifelong music participation, with implications for multigenerational collaboration within school music programs. The third presentation extends the theme of social connection by introducing research on the use of silent disco headphones in long-term care settings, demonstrating their potential to cultivate immersive, shared musical experiences that enhance joy and engagement. Shifting toward a clinical lens, the final presentation explores group drumming as a non-pharmacological intervention to support well-being, inclusion, and participation among older adults with intellectual and developmental disabilities. A concluding discussion synthesizes these findings, highlighting key intersections and inviting attendees to contribute insights on reimagining music’s role in aging. Attendees are encouraged to take the ideas and insights from this symposium back to their own context and reimagine new possibilities in using music to promote the well-being of older adults and the broader community.

